# Multi-breed genome-wide association studies across countries for electronically recorded behavior traits in local dual-purpose cows

**DOI:** 10.1371/journal.pone.0221973

**Published:** 2019-10-30

**Authors:** Tong Yin, Maria Jaeger, Carsten Scheper, Gregorz Grodkowski, Tomasz Sakowski, Marija Klopčič, Beat Bapst, Sven König

**Affiliations:** 1 Institute of Animal Breeding and Genetics, Justus-Liebig-University of Gießen, Gießen, Germany; 2 Institute of Genetics and Animal Breeding of the Polish Academy of Sciences, Jastrzębiec, Poland; 3 University of Ljubljana, Biotechnical Faculty, Department of Animal Science, Domzale, Slovenia; 4 Genetic evaluation center, Qualitas AG, Switzerland; The University of North Carolina at Chapel Hill, UNITED STATES

## Abstract

Basic bovine behavior is a crucial parameter influencing cattle domestication. In addition, behavior has an impact on cattle productivity, welfare and adaptation. The aim of the present study was to infer quantitative genetic and genomic mechanisms contributing to natural dual-purpose cow behavior in grazing systems. In this regard, we genotyped five dual-purpose breeds for a dense SNP marker panel from four different European countries. All cows from the across-country study were equipped with the same electronic recording devices. In this regard, we analyzed 97,049 longitudinal sensor behavior observations from 319 local dual-purpose cows for rumination, feeding, basic activity, high active, not active and ear temperature. According to the specific sensor behaviors and following a welfare protocol, we computed two different welfare indices. For genomic breed characterizations and multi-breed genome-wide association studies, sensor traits and test-day production records were merged with 35,826 SNP markers per cow. For the estimation of variance components, we used the pedigree relationship matrix and a combined similarity matrix that simultaneously included both pedigree and genotypes. Heritabilities for feeding, high active and not active were in a moderate range from 0.16 to 0.20. Estimates were very similar from both relationship matrix-modeling approaches and had quite small standard errors. Heritabilities for the remaining sensor traits (feeding, basic activity, ear temperature) and welfare indices were lower than 0.09. Five significant SNPs on chromosomes 11, 17, 27 and 29 were associated with rumination, and two different SNPs significantly influenced the sensor traits “not active” (chromosome 13) and “feeding” (chromosome 23). Gene annotation analyses inferred 22 potential candidate genes with a false discovery rate lower than 20%, mostly associated with rumination (13 genes) and feeding (8 genes). Mendelian randomization based on genomic variants (i.e., the instrumental variables) was used to infer causal inference between an exposure and an outcome. Significant regression coefficients among behavior traits indicate that all specific behavioral mechanisms contribute to similar physiological processes. The regression coefficients of rumination and feeding on milk yield were 0.10 kg/% and 0.12 kg/%, respectively, indicating their positive influence on dual-purpose cow productivity. Genomically, an improved welfare behavior of grazing cattle, i.e., a higher score for welfare indices, was significantly associated with increased fat and protein percentages.

## Introduction

The current fundamental interest in dairy cattle research addresses a deeper understanding of the role of genetics in phenotypic expressions of behavior traits. Behavior is an essential part of biological regulations and influences the production and welfare of farm animals. However, the underlying genetic mechanisms explaining the relationships between cattle behavior and productivity are unclear. Currently, consumer demands strongly influence animal husbandry and management decisions, e.g., a wish towards the utilization of natural and cow friendly production systems. Furthermore, there are increasing concerns, critically addressing the high yielding Holstein Friesian breed and suggesting local dual-purpose cattle as a breed alternative. Against this background, a better understanding of the genetic mechanisms of animal behavior allows for the implementation of local dual-purpose cattle selection strategies for specific environments, e.g., for specific grazing conditions. Hohenboken [1] listed behavior traits in cattle under genetic control, such as feeding and reproductive behavior, social interactions and temperament. In addition, especially in grazing systems, a proportion of variation in foraging behavior is genetically inherited [2,3]. In addition to feeding, rumination time and rumination intervals are defined as novel traits that influence milk yield and butterfat production [4]. Nevertheless, subjectively scored cattle behavior traits are low to moderate heritability traits, with heritabilities ranging from 0.01 to 0.44 [5,6]. Despite a few quantitative genetic studies based on pedigree relationship matrices, there is a gap in knowledge addressing genomic mechanisms of behavior trait expressions [4]. Dense longitudinal phenotypic data and dense single nucleotide polymorphism (SNP) marker information are required to perform genome-wide association studies (GWAS) and to unravel the genetic architectures of complex traits. Consequently, only a limited number of potential candidate genes significantly associated with cattle behavior traits were identified [7]. Alam et al. [8] detected polymorphisms of the bovine neuropeptide Y5 receptor gene (*NPY5R*), which regulates appetite and feeding behavior in beef cattle. Similar mechanisms for polymorphisms of the melanocortin 4 receptor gene (*MC4R*), i.e., influences on feed intake capacity and feeding behavior, were reported in Korean Hanwoo cattle [9]. Nevertheless, a strong environmental component influences behavior trait expressions, suggesting a detailed recording of environmental effects for a broad pattern of behavior characteristics [4].

In the process of animal husbandry system intensifications, domestication and artificial selection via specific mating plans were major driving components contributing to extensive linkage disequilibrium (LD) across the bovine genome [10,11]. Consequently, broad confidence intervals for significant SNP were identified, implying difficulties in precisely mapping potential candidate genes [10]. Raven et al. [10] hypothesized that lower levels of long-range LD across bovine breeds, and thus, a multi-breed GWAS, could accurately pinpoint the location of well-conserved functional mutations. When considering several breeds simultaneously, LD over short distances (5–10 kb for *Bos taurus*) already reached r^2^ > 0.3 [12], while long-range LD decreased. Hence, with higher probability compared to a single-breed GWAS, a significant SNP from a multi-breed GWAS is located in close distance to a quantitative trait locus (QTL), which has an effect on the same trait across breeds. Significant across-breed SNP effects are mainly due to LD with the QTL and are independent of pedigree relationship influences [11,13]. This phenomenon is well exploited in refining QTL regions in dogs, but the methodology only contributed to a limited number of identified potential candidate genes [14]. In detail, in the dog study, identification of QTLs was based on a single dog breed with extensive LD. In a second step, multiple dog breeds and dense SNP chips were used to precisely map causal variants [10,14]. Hence, with regard to QTLs segregating in multiple breeds, a multi-breed GWAS implies more precise mapping, while within-breed analyses contribute to improved detection power for breed-specific QTLs. Hence, a multi-breed GWAS might increase the probability of detecting older conserved mutations, but it is less efficient in identifying recently diverged mutations [10]. With the aim of inferring the causes of general and well-conserved genetic mechanisms in basic bovine behavior traits, a multi-breed GWAS seems to be a promising method.

The current study is based on SNP data from five dual-purpose cattle breeds located in Germany (DE_DSN = black and white dual-purpose cattle), Poland (PL_BS = Brown Swiss, PL_DSN = black and white dual-purpose cattle), Slovenia (Sl_BS = Brown Swiss, Sl_Si = Simmental) and Switzerland (CH_OBV = dual-purpose Original Braunvieh, CH_Si = Simmental). Genotyped cows were phenotyped based on 24 hours of continuously recorded behavior data in grazing systems. The overall hypothesis is that electronically recorded natural behavior of cows for feeding (FEED), ruminating (RUM), resting / non-active (NACT), basic activity (BACT) and high activity (HACT) and digital ear surface temperature (ET) contributes to the detection of significant SNP markers and associated potential candidate genes across the bovine genome. Additionally, for population structure analyses, we considered genotypes from the dual-purpose Red and White breed from Germany (DE_DN) and from German Holstein (DE_HF) and Slovenian Holstein (Sl_HF) subpopulations. We assume that different breeds with a similar breeding history share ancestral mutations and recombination events. Accordingly, Gutiérrez-Gil et al. [15] identified selection signatures influencing metabolic homeostasis and disease resistance across breeds with different production trait characteristics.

The present study is based on dense genomic marker data and longitudinal behavior traits from different dual-purpose cows across European country borders. Such unique data can be used i) to infer the population structure for European dual-purpose and dairy cattle breeds; ii) to estimate genetic parameters for behavior traits based on pedigree and genomic information; iii) to detect associated SNP and potential candidate genes significantly influencing cattle behavior; and iv) to infer causal trait associations.

## Results

### Population structure and breed assignment

#### Principal component analyses

**Fig 1:**
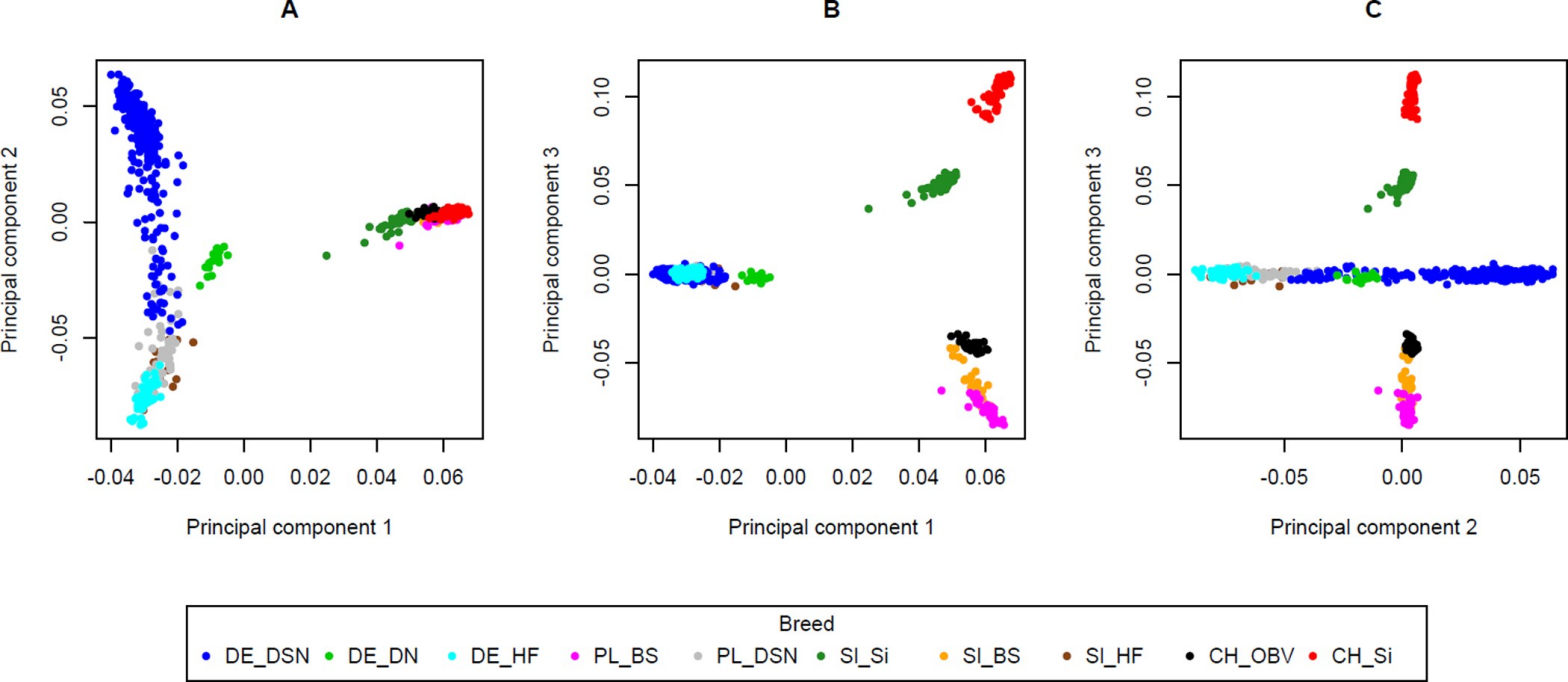
Plot of principal components 1 versus 2 (A), 1 versus 3 (B) and 2 versus 3 for the genomic relationship matrix based on 615 genotyped cows. DE_DSN = black and white dual-purpose (Germany); DE_DN = red and white dual-purpose (Germany); DE_HF = Holstein Friesian (Germany); PL_BS = Brown Swiss (Poland); PL_DSN = black and white dual-purpose (Poland); Sl_Si = Simmental (Slovenia); Sl_BS = Brown Swiss (Slovenia); Sl_HF = Holstein Friesian (Slovenia); CH_OBV = dual-purpose Original Braunvieh (Switzerland); CH_Si = Simmental (Switzerland).

When plotting the first and the second principal components (explaining 4.71% and 3.05% of the variation in genomic relationships, respectively), two distinctly diverged clusters of genetic origin were detected (Fig 1A). The Holstein lines and DSN showed obvious genetic differentiation from the other breeds (Sl_Si, Sl_BS, PL_BS, CH_OBV, and CH_Si). Depicting the first and third (explaining 2.38% of variation) principal components, three clusters were formed in a triangle-like 2-dimensional form (Fig 1B). Each cluster was positioned at the three apexes of the triangle, with the admixed populations of Sl_Si in an intermediate position. The first cluster includes DE_HF, DE_DSN, DE_DN, Sl_HF and PL_DSN; the second cluster consists of PL_BS, Sl_BS and CH_OBV; and CH_Si and Sl_Si (but in slight distance) are represented in cluster 3. The three clusters were also identified when plotting the second and third principal components (Fig 1C). However, the second principal component illustrates the diversity within the Holstein lines and DSN.

#### Breed assignment

**Fig 2:**
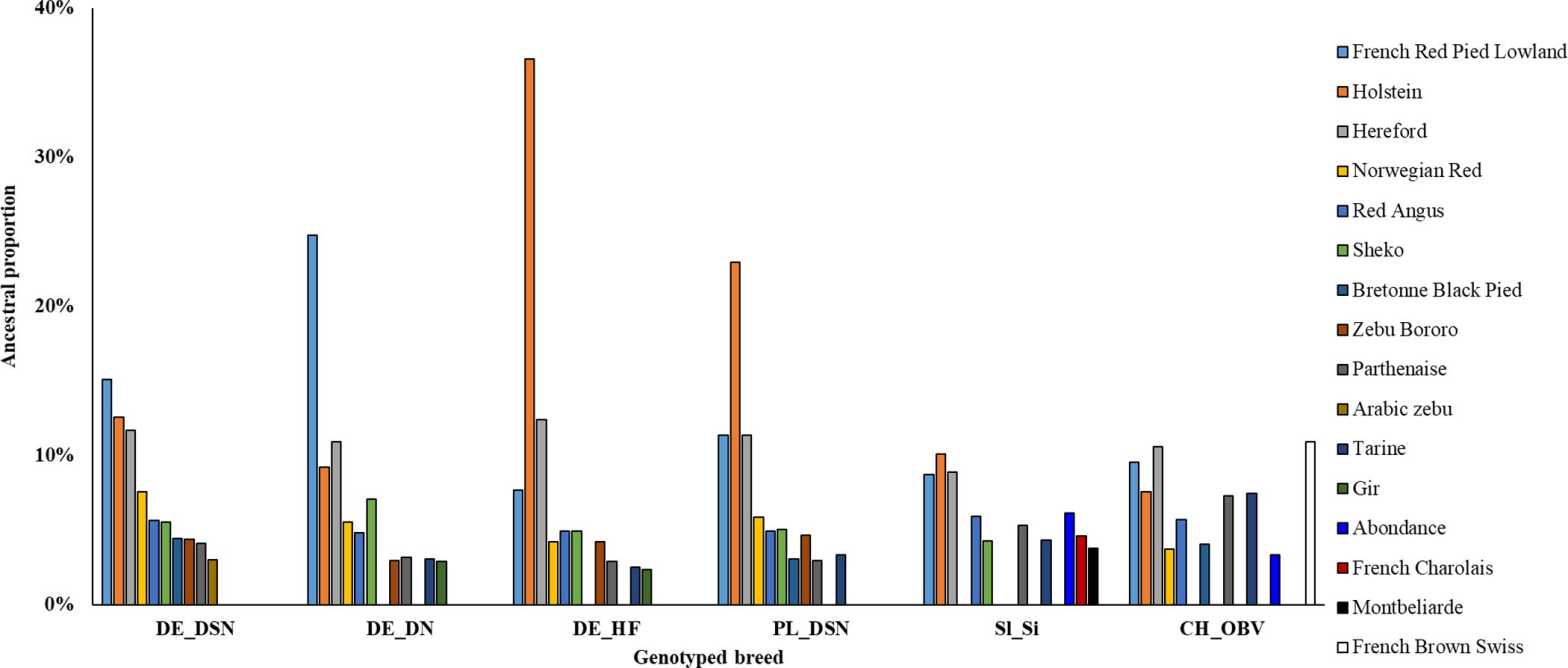
Ancestry composition of the genotyped cows considering the ten reference populations in WIDDE [16]. DE_DSN = black and white dual-purpose (Germany); DE_DN = red and white dual-purpose (Germany); DE_HF = Holstein Friesian (Germany); PL_DSN = black and white dual-purpose (Poland); Sl_Si = Simmental (Slovenia); CH_OBV = dual-purpose Original Braunvieh (Switzerland).

The breed assignment (Fig 2) identified ten cattle breeds with the largest ancestry proportions from the world reference dataset in Web-Interfaced Next Generation Database (WIDDE) [16] for the populations in this study. All populations from our study shared at least 57.83% of alleles with European breeds, affirming their European origin. The predominant genetic ancestry consisted of Holstein, Hereford, French Red Pied Lowland and French Brown Swiss breeds. However, aside from European ancestors, exotic ancestral proportions from Sheko, Zebu Bororo, Gir or Arabic Zebu appeared.

### Genetic parameters for sensor traits

Heritabilities and corresponding standard errors or sensor traits were on a low to moderate level but very similar for the estimations based on either the pedigree relationship matrix (**A**) or the combined relationship matrix (**H**) matrices (Table 1). Heritabilities for FEED, HACT and NACT ranged from 0.16 to 0.20 due to moderate additive genetic and small residual variances. The low heritable traits RUM, BACT, ET, welfare index points (WEL-IP) and welfare index classes (WEL-IC) had small additive genetic variances. Repeatabilities were moderate (0.24–0.34) for sensor traits but ranged on a lower level from 0.10 to 0.13 for the two welfare indices. Standard errors of repeatabilities from the multi-breed estimation were quite small (0.01–0.02).

**Table 1:** Heritabilties (h^2^) and reliabilities (r) with standard errors (SE) for sensor behavior traits.

	Pedigree				Combined pedigree and genomic data			
**Trait**	h^2^	SE	r	SE	h^2^	SE	r	SE
**RUM**	0.02	0.04	0.28	0.02	0.02	0.04	0.28	0.02
**FEED**	0.19	0.05	0.26	0.02	0.20	0.05	0.27	0.02
**BACT**	0.08	0.05	0.26	0.02	0.06	0.05	0.26	0.02
**HACT**	0.19	0.05	0.27	0.02	0.20	0.05	0.29	0.02
**NACT**	0.16	0.06	0.33	0.02	0.18	0.06	0.34	0.02
**ET**	0.07	0.04	0.24	0.02	0.07	0.04	0.25	0.02
**WEL-IP**	0.03	0.02	0.12	0.01	0.04	0.02	0.13	0.01
**WEL-IC**	0.03	0.02	0.10	0.01	0.04	0.02	0.10	0.01

RUM = rumination; FEED = feeding; BACT = basic active; HACT = high active; NACT = not active; ET = ear temperature; WEL-IP = welfare index point; WEL-IC = welfare index class.

### Multi-breed genome-wide association study

Overall, according to the 20% false discovery rate (FDR) threshold, seven SNP markers were significantly associated with behavior traits (Table 2). One of these SNPs was also significant considering the more stringent Bonferroni threshold. Significant SNPs were located on six different chromosomes and associated with the behavior traits NACT, RUM and FEED. The most significant SNP influencing NACT is located on *Bos taurus* autosome 13 (BTA13, *P-value =* 2.36E-08) (S1 Fig). Five SNPs on BTA11, 17, 27, and 29 were significantly associated with RUM (S2 Fig), and another SNP on BTA23 was significantly associated with FEED (S3 Fig). A more significant SNP was detected for the dependent variable de-regressed proof (DRP) in comparison to the means of repeated sensor traits (MEAN). Only for NACT, the same SNP on BTA13 (Hapmap60738-rs29023086) significantly influenced both dependent variables MEAN and DRP. The inflation factors for all GWAS runs ranged from 1.00 to 1.04 for DRP, and from 0.88 to 0.99 for MEAN, indicating restricted false positives from population stratification.

**Table 2:** List of SNP markers significantly associated with sensor behavior traits (significance threshold: False discovery rate of 20%).

Trait	Chr.	SNP	bp	*P-value*	Method	Prop.
**RUM**	11	BTB-01638234	55229674	2.04E-05	DRP	2.73%
**RUM**	17	ARS-BFGL-NGS-104430	68187177	1.79E-05	DRP	3.38%
**RUM**	27	ARS-BFGL-NGS-13449	37283994	1.36E-05	DRP	3.03%
**RUM**	29	ARS-BFGL-NGS-24800	46014507	9.07E-06	DRP	2.91%
**RUM**	29	ARS-BFGL-NGS-81862	49036580	2.01E-05	DRP	3.43%
**FEED**	23	ARS-BFGL-NGS-80066	19834215	5.13E-06	DRP	4.41%
**NACT**	13	Hapmap60738-rs29023086	79178395	2.36E-08 1.08E-06	DRP MEAN	5.51% 3.63%

RUM = rumination; FEED = feeding; NACT = not active; Chr. = chromosome number; bp = base pair; DRP = de-regressed proof; MEAN = mean of observations; Prop. = proportion of phenotypic variance explained by SNP markers.

The SNP coverage was examined by counting the number of SNPs in consecutive windows of 1 Mb on each chromosome. The mean SNP coverage per Mb considering the 29 autosomes was 14.2, ranging from zero to 27 SNP per Mb. At least one Mb window without a SNP was identified on ten chromosomes (BTA6, 7, 10, 12, 14, 15, 18, 21, 24 and 26).

With regard to the GWAS for fat percentage (Fat%), two significant SNPs above the Bonferroni corrected threshold on BTA14 (S4 Fig) were identified. This is the chromosomal segment for the *DGAT1* gene. Hence, the multi-breed GWAS identified the most prominent candidate gene in dairy and dual-purpose cattle.

### Potential candidate genes

Based on the *P-values* of all SNPs, i.e., GWAS output, 22 potential candidate genes were identified (S1 Table). All of the inferred potential candidate genes might play a role in the expression of bovine behavior in dual-purpose cattle populations. The 13 potential candidate genes on BAT21, 27, and 29 are associated with RUM. For RUM, one potential candidate gene (*BTBD1*) is located on BAT21, and two are on at BAT27 (*THAP1* and *RNF170*). The remaining ten potential candidate genes are located on at BAT29. Both dependent variables for RUM (DRP and MEAN) were associated with eight putative candidate genes (*RPS6KB2*, *PTPRCAP*, *CORO1B*, *GPR152*, *CABP4*, *TMEM134*, *AIP*, and *PITPNM1*). With regard to the DRP for RUM, we identified two potential candidate genes without clear biological functions on BTA29 (the novel gene *ENSBTAG00000000776* and *MRGPRG)*. The SNP Hapmap48998-BTA-104140 (*P-value* = 6.55E-06 on BAT19) suggested *PPM1E* as a potential candidate gene for BACT. With regard to the DRP of FEED, we identified seven potential candidate genes on BAT11 (*STXBP1*, *CFAP157*, *PTRH1*, *TOR2A*, *LCN8*, *LCN15* and *PPP1R26*). The two neighboring SNPs ARS-BFGL-NGS-80066 and ARS-BFGL-NGS-111955 on BTA23 indicated one putative candidate gene (*SLC25A27*) for FEED.

### Causal associations

The regression coefficients for the variety of trait associations are summarized in Table 3. The values in bold indicate significance according to FDR < 0.05. Behavior related to feed intake, i.e., RUM and FEED, had a significantly negative impact on behavior reflecting locomotion (BACT and HACT) and vice versa. Associations were positive between traits from the same behavior category, i.e., between FEED and RUM, and between HACT and BACT. For example, for an increase of 1% in FEED, RUM increased by 0.12%. The regression coefficient of HACT on BACT was 0.18%. Sleeping and resting (NACT) negatively influenced RUM, FEED, and HACT. When NACT was the exposure and BACT was the outcome, the slope was 0.10%. The impact of behavior traits on ET was generally weak and not significant. For example, a 1% increase in HACT was associated with an increase of 0.11 degrees Celsius for ET. Apart from HACT, behavior traits responded significantly to alterations of welfare indices because behavior traits are components of the indices. An increase in feeding behavior (RUM and FEED) had favorable effects on milk yield (MY) but impaired udder health (increase of somatic cell score; SCS). A regression coefficient of -0.14 kg/% was estimated for the response of MY on NACT. Welfare indices were significantly associated with Fat% and protein percentage (Pro%).

**Table 3:** Regression coefficients among sensor behavior traits and between sensor behavior traits and production traits.

Trait	RUM	FEED	BACT	HACT	NACT	ET	WEL-IP	WEL-IC	MY	Fat%	Pro%	SCS
**RUM**		**0.14**	**-0.40**	**-0.09**	**-0.51**	0.02	0.02	**0.00**	0.10	0.00	0.00	**0.05**
**FEED**	**0.12**		**-0.34**	**-0.06**	**-0.48**	0.00	**-0.05**	**0.01**	**0.12**	0.00	0.00	0.02
**BACT**	**-0.77**	**-0.78**		**0.18**	0.19	-0.01	**0.05**	**-0.02**	-0.14	0.00	0.00	-0.05
**HACT**	**-0.51**	-0.29	**0.59**		**-0.78**	0.11	**0.07**	-0.01	0.04	0.02	0.01	-0.05
**NACT**	**-0.41**	**-0.59**	**0.10**	**-0.10**		-0.01	**-0.03**	**0.01**	**-0.14**	0.00	0.00	-0.02
**WEL-IP**	**1.60**	**-0.97**	**0.80**	0.29	**-1.72**	**0.44**		**-0.23**	-0.10	0.07	**0.05**	0.12
**WEL-IC**	**-5.00**	1.74	**-4.67**	-0.61	**7.99**	-1.44	-3.56		1.40	**-0.57**	**-0.30**	-0.65

RUM = rumination; FEED = feeding; BACT = basic active; HACT = high active; NACT = not active; ET = ear temperature; WEL-IP = welfare index point; WEL-IC = welfare index class; MY = milk yield (in kg); Fat% = fat percentage; Pro% = protein percentage; SCS = somatic cell score; the bold values represent significant regression coefficients.

## Discussion

### Population structure analyses

The identified breed clusters from the principal component analysis (PCA) reflect the geographical origin of the European cattle breeds. The optimization criterion for PCA is the maximization of variation in the genomic relationship matrix considering the first principal components [17], which also contribute to geographic differentiation. The PCA clearly differentiated between the Holstein lines and DSN with the Simmental and Brown Swiss breeds (Fig 1A), regardless of geographic distance. Hence, these breeds have different ancestors and do not share the same founder alleles. Due to their pronounced genetic relationships, Sl_HF, PL_DSN, DE_HF, DE_DSN, and DE_DN were allocated to the same cluster. The other two distinct clusters represent Simmental and Brown Swiss breeds (Fig 1B and 1C). The origin of genotypes certainly contributed to cluster formation. Furthermore, breed-specific breeding goals or country and farm specificities influenced breed differentiations [18]. Interestingly, only the second principal component presented genetic diversity within the cluster containing Holstein lines and DSN. Because of different breeding goal definitions [19], commercial Holstein lines (DE_HF and Sl_HF) were separated from black and white dual-purpose cattle (DE_DSN and DE_DN) during selection. The PL_DSN, reflecting an intermediate breeding goal “between” classical dual-purpose cattle and modern HF, is consequently grouped between Holstein lines and DSN. Nevertheless, DSN is the dominant founder population for modern HF [20], and similar breeding schemes and an identical herdbook were used before officially separating the two breeds in 1997 [21]. Differentiations between the Holstein lines and DSN underline the footprints of artificial selection in the last two decades [22]. Although DE_DN and DE_DSN are dual-purpose breeds, they share ancestors with Holstein Friesian cattle more than 50 years ago, explaining their rather close relationship. As a consequence, the PCA results reflect these breed origins and separate endogamous breeding units for Holstein, DSN, Brown Swiss and Simmental cattle, emphasizing the historical ‘genetic isolation’ by the absence of admixture.

Breed assignment results gave deeper insights into the pattern of genetic diversity and principles of historical evolutionary processes in dual-purpose cattle populations. All breeds depicted at least 57.83% of ancestral allele proportion to European cattle breeds, affirming their European origin. Nevertheless, for the European dual-purpose cattle genotypes, exotic ancestral allele proportions from Sheko, Zebu Bororo, Gir or Arabic Zebu were identified. Despite the fact that cattle are ascribed to two major geographic types, i.e., taurine (humpless- European, African, Asian) and indicine (humped- South Asian, East African), the same ancestors were identified more than 250,000 years ago [12]. Ancient genetic ties to a common ancestor as well as interbreeding [12] explain a proportion of up to 7.09% shared alleles between Sheko with DE_DSN, DE_DN, DE_HF, PL_DSN and Sl_Si. In this regard, the Bovine HapMap Consortium [12] specified five unique endogamous breeding units (Holstein, Jersey, Hereford, Romagnola, and Guernsey) and one closed endogamous breeding unit (Brown Swiss, Norwegian Red, Limousin, Charolais, and Piedmontese) for ten European breeds. Furthermore, they [12] identified indicine and taurine crosses, such as Beefmaster, Santa Gertrudis and Sheko. Accordingly, in the present study, low proportions of common ancestry between populations from our study and indicine breeds were identified.

Early breeders who spread from the Fertile Crescent towards North-West Europe used two different migration routes [23]. One route to northern Europe followed the Balkan rivers (Danubian route) to Germany and the Netherlands, while the second route (Mediterranean route) to western Europe (Italy, Spain and France) crossed the Mediterranean Sea [23]. During these migration waves, potential interbreeding between wild European aurochs and already domesticated populations explain the exotic breed footprints within the European bovine genome [17]. These findings are in agreement with the known shared ancestry between Holstein and Norwegian Red [12]. Consequently, we also detected genetic relations between DE_DSN, DE_DN, DE_HF and PL_DSN with Norwegian Red. Gautier et al. [17] affirmed the Northern European origin of Angus, Red Angus, French and American Holstein, French Red Pied Lowland and Norwegian Red cattle via Reynolds genetic distances (computation based on allele frequencies at 44,706 SNP loci). Hence, these results [17] support the identified ancestry proportions, as illustrated in Fig 2. Close genetic proximity between French Red Pied Lowland with DE_DSN, DE_DN, and DE_HF is due to the Red Pied Lowland’s recent derivation from Red Holstein and Meuse-Rhin-Yssel breeds [17]. Relatively high proportions of ancestry between Hereford with Sl_Si (8.87%), CH_OBV (10.58%), DE_DN (10.92%), PL_DSN (11.37%), DE_DSN (11.69%) and DE_HF (12.39%) were identified. Accordingly, Gautier et al. [17] allocated Hereford, Holstein and Brown Swiss to one major cluster. The genetic influence of Hereford on Holstein, DSN, Brown Swiss and Simmental genomes is due to historical interbreeding events [24], which occurred before the establishment of the Hereford breed herd in 1846 [25].

The PCA results as well as the breed assignment analyses indicate the European origin of dairy (DE_HF) and dual-purpose breeds (DE_DSN, DE_DN, PL_DSN, Sl_Si, and CH_OBV) and reflect selection according to geographic and breeding goal characteristics. The evolutionary formative events contributed to the establishment of different genetic variants in cattle breeds in different regions. Moreover, they influenced the differentiation of allele frequencies among populations [12] and the associations between phenotypes and genotypes.

### Genetic parameters for sensor behavior traits

Apart from BACT, genetic parameter estimates from the pedigree-based approach (**A** matrix) were very similar or slightly smaller compared to the **H** matrix approach (i.e., additionally considering genomic marker data). This result is in agreement with other studies focusing on genetic parameter estimations based on different genetic relationship matrices [26,27]. Basic dairy cattle habits (e.g., HACT, NACT and FEED) underlie moderate genetic control. For RUM, BACT, ET, WEL-IP and WEL-IC, the small heritabilities indicate pronounced environmental influence and challenges for genetic improvements. Nevertheless, the recording technique might also explain the lower heritabilities for sensor-recorded RUM and BACT. As a recording alternative, microphone-monitored rumination time contributed to heritability estimates for RUM in the range from 0.14 to 0.44 in Holstein cows [6]. Another reason for the smaller RUM heritability in the current study addresses characteristic differences in the production system. In grazing systems, with a higher percentage of fresh fibrous grass in the feeding ratio, rumination mechanisms might differ from total mixed rations fed in indoor systems. Hence, the genetic mechanisms for rumination might differ and influence additive genetic variances. A strong impact of food characteristics on rumination time was identified in previous studies, e.g., the influence of forage neutral detergent fiber [28], physical effective fiber [29], or long-particle alfalfa silage [30]. Nevertheless, rumination time is an interesting trait for genomic selection because of the moderate to strong association with feed efficiency [6,31]. Feeding costs are the dominant cost component in dairy and dual-purpose cattle farming systems [32]. Consequently, the selection of RUM also contributes to high feed efficiency and profitable milk production [33].

The heritability for FEED behavior is in agreement with estimates from other studies using alternative recording techniques. Løvendahl and Munksgaard [5] estimated a heritability of 0.20 for pooled eating time (hour/day) considering early and late lactation stages. Eating time was recorded via focal scanning in batches at 10-minute intervals for 24 hours. Robinson and Oddy [34] reported a heritability of 0.36 for time spent feeding, measured in automatic feeder pens. Hence, feeding time has a moderate genetic component, but the open question addresses the optimal breeding and selection strategy. A breeding goal with a focus on increasing feeding time (FEED) implies an antagonistic impact on other types of behavior, e.g., reduced lying time (NACT) [35,36].

Heritabilities for daily BACT reflect estimates based on accelerometer recordings (0.03–0.12) [37,38]. Schöpke and Weigel [37] considered 1,171 postpartum HF cows with at least 100 days of consecutive accelerometer measurements, and the HACT accelerometer heritabilities support the HACT sensor heritabilties from our study. Furthermore, in agreement with our results, variance components and heritabilities were different for different levels of activity, i.e., during nonestrus periods in the range of 0.03–0.05 and 0.12 during estrus [37]. Coincidently, in our study, heritabilities for HACT were larger than for other behavior activities. Nevertheless, the NACT heritability from the cows in the outdoor grazing system was larger than the heritability estimates of dairy cattle for lying time indoors (0.01) [5]. Even in humans, genetic parameters for active and non-active behavior traits have been estimated. Our heritability estimate for lying or sleeping is in agreement with the heritability for children sleeping duration [39]. A quite larger heritability was estimated for total daily sleep duration (daytime sleep duration plus nighttime sleep duration), considering 53 pairs of monozygotic and dizygotic female twins [40]. However, such an estimate might be biased due to a large proportion of common environmental effects in twins’ samples.

The low heritability estimates for ET indicate partial genetic control of temperature regulation mechanisms but a stronger impact due to environmental effects and production levels [41]. Heritabilities for rectal temperature were larger in the range from 0.15 to 0.17 [41,42]. Nevertheless, regarding trait definition, there is a difference when measuring surface or rectal temperature [42]. Environmental temperature had a stronger impact on surface ET than on rectal and core body temperature [42,43]. Hence, heritabilities for rectal temperature were larger in the range from 0.15 to 0.17 [41,44]. The complex definition of welfare indices and the inclusion of several antagonistic related traits might explain the quite small heritabilities and repeatabilities for WEL-IP and WEL-IC. In conclusion, we suggest the utilization of welfare indices as a novel management tool and not as a selection instrument to improve an animal’s welfare status via breeding.

### Multi-breed genome-wide association study

To our knowledge, this is the first study considering dense sequences of longitudinal behavior measurements of dual-purpose cows from grazing systems across countries, combined with high-throughput genomic marker data. On the basis of a multi-breed GWAS, we gained new insights into the genetic control of dual-purpose cattle behavior under grazing conditions, and we located some interesting chromosomal segments. Nevertheless, for the detection of causal functional mutations in ongoing studies, it is imperative to use denser SNP data or even sequence data and a larger sample of genotyped cows [45,46]. Regarding the response traits, DRP reflected the daily behavior expression more accurately than one single MEAN value. In the statistical models for DRP, all important environmental (fixed) effects influencing bovine behavior [47] were considered. For the dependent variable MEAN, pre-correction of the data only accounted for the ‘breed-farm’ effect. Consequently, we suppose that the MEAN from an extended observation period does not fully reflect the genetic variation of bovine behavior due to confounding environmental effects. Additionally, when referring to the multi-breed GWAS, only one significant SNP was detected via the MEAN approach, but seven significant SNPs were discovered using DRP.

The identification of the *DGAT1* gene on BTA14 supported our a priori hypothesis that (despite the small sample size) the multi-breed GWAS is an appropriate approach to identify putative causative variants and candidate genes. Using an FDR of 20%, the number of identified significant genetic variants, including SNP and potential candidate genes, was larger compared to the stricter Bonferroni correction. However, the risk of detecting false positive SNPs also increased. The consideration of accumulated effects from a set of SNPs ±50 Kb of a gene (set-based association) was very powerful for detecting potential candidate genes, as suggested in previous studies [48]. Some of the discovered potential candidate genes are linked to behavior traits or diseases in cattle [49], humans [50], pigs [51] or mice [52].

#### Rumination

Based on the five significant SNPs with FDR < 20%, we detected 13 potential candidate genes for RUM. Mutations of the identified potential candidate gene *RNF170* were associated with autosomal dominant sensory ataxia in humans [53]. The putative candidate gene *RPS6KB2* is involved in innate immune response mechanisms in indigenous and crossbred cattle [54]. In addition, the gene *RPS6KB2* was differentially expressed in Angus cattle selected for low and high residual feed intake [49] and in bovine tuberculosis-infected and control cattle [55]. Other findings suggest an association of *RPS6KB2* with embryonic development in cattle [56]. The *PTPRCAP* gene is an additional identified potential candidate gene that is associated with RUM behavior. In humans, *PTPRCAP* is involved in defense response mechanisms and is a key regulator of lymphocyte activation [50].

The putative candidate gene *CaBP4*, coding for a neuronal Ca2+-binding protein, was expressed in photoreceptors in mice and regulated synaptic terminals [52]. Haeseleer et al. [52] concluded that *CaBP4*−/− mice have behaviors similar to those in patients with incomplete congenital stationary night blindness. Generally, *CaBP4* is involved in the process of signal transduction [57] and visual perception [58].

The identified potential candidate gene *TMEM134* influences obesity and atherosclerosis in adults [59]. Furthermore, *TMEM134* is involved in the prototypical inflammatory nuclear factor-κB (*NF-κB*) signaling pathway [59]. The modulation of downstream *NF-κB* signaling is the most important characteristic for innate immune programming in chronic inflammation [60]. The identified potential candidate gene *PITPNM1* is associated with retinal degeneration and hypopyon in humans and is involved in pathways of metabolism and glycerophospholipid biosynthesis [61].

#### Feeding

The potential candidate gene *LCN15* for FEED is involved in the transport of glucose and other sugars, bile salts and organic acids, metal ions and amine compounds as well as the transport of vitamins and nucleosides [61]. As a member of the lipocalin gene family, *LCN2* influences obesity and diabetes in humans [62]. Furthermore, *LCN15* physiologically interacted with high glucose levels in enterocytes [62]. Extended periods for FEED indicate an increase in feed intake [34], implying higher levels of sugars, fatty acids, amino acids, and vitamins. Hence, cows with different FEED levels might differ regarding specific expression profiles for the potential candidate gene (*LCN15*).

The potential candidate gene *SLC25A27* is part of a recently identified genetic network associated with economically important traits in Wagyu x Limousin crossbred cattle [63]. Additionally, *SLC25A27* contributes to long chain fatty acid uptake [63] and controls several diseases in humans, such as Alzheimer’s disease [64], oxidative stress [65], and fasting [66]. The mitochondrial uncoupling protein 4 encoded by the *SLC25A27* gene is involved in thermoregulatory heat production and metabolism in the brain [67].

#### Basic activity

Only one potential candidate gene (*PPM1E*) influenced BACT behavior in dual-purpose cattle. Accordingly, the dephosphorylation gene (*PPM1E*) was associated with feeding behavior in Danish Duroc boars [51]. Do et al. [51] assumed that *PPM1E* is mediated by 5’AMP-activated protein kinase (*AMPK*), which plays a key role in controlling energy balances. The enzyme *AMPK* is involved in hypothalamic glucose and nutrient sensing. Hence, due to the identified impact of *PPM1E* on activity traits in dual-purpose cattle and due to the strong correlation between feeding and activity (S2 Table), behavior across species is based on the same genetic mechanisms.

### Associations among behavior traits and between behavior and productivity

The behavior traits RUM, FEED, BACT, HACT and NACT were interdependent, implying that the expression of basic behavior is involved in similar physiological processes. Additionally, from a practical perspective, some strong associations were expected. For example, an increase of feed intake (FEED) implies intensification of rumination time (RUM). Behavior-related feed intake had negative genetic impacts on BACT, enhanced BACT (HACT), and resting/sleeping (NACT) [68]. An increase in rumination and feeding contributes to improved milk production [69], but intensification of “production behavior” implies less time for BACT, HACT and NACT. “Normal” daily BACT behavior of dual-purpose cows was in balance with sleeping behavior (NACT). However, during estrus or parturition, cows express excessive walking, mounting and overall restlessness behavior (HACT), while the usual resting habits decrease [70] and body temperature increases [71,72]. Interestingly, welfare indices were also associated with ET.

Cows with 1% higher levels for RUM and FEED produced 0.10 kg and 0.12 kg more milk [68], respectively, along with increased somatic cell count. A simultaneous increase of SCS is due to the antagonistic relationship between MY and SCS [73]. High levels of daily BACT positively correlated with body condition loss, implying a reduction in MY [74]. The positive impact of BACT on NACT might explain the negative regression coefficient of NACT on MY. In general, daily bovine behaviors, including RUM, FEED, BACT, HACT, and NACT, do not have a significant impact on Fat% and Pro%. However, improved welfare indices were associated with higher values for Fat% and Pro%. Currently, in practical breeding schemes, Fat% and Pro% are used as indicators to assess the cows’ energy status [75]. Additionally, based on the results from the present study, Fat% and Pro% might be suitable indicator traits for cattle welfare.

## Materials and methods

### Animal ethics statement

Genotype data were provided from the national breeding organizations. Phenotypes reflect the standard trait pattern from official milk recording schemes. Behavior recording was non-invasive.

### Breeds and herd location

The five dual-purpose cattle breeds with phenotypic sensor behavior data were from Germany (DE_DSN), Poland (PL_BS, PL_DSN), Slovenia (Sl_BS, Sl_Si) and Switzerland (CH_OBV, CH_Si) (Table 4). In Germany and Poland, dual-purpose cows were kept in organic university research herds. The German research farm belongs to the federal state of Hesse in the center of Germany, and the farm in Poland is close to the Baltic Sea. In Slovenia, data recording considered three commercial grazing herds located in mountainous regions in the western part of the country, at 920 m to 970 m above sea level. In Switzerland, one original Braunvieh near Lucerne and one Simmental herd in the region around Basel were chosen for the across-country analyses. All farming conditions reflect pasture-based production systems, allowing grazing for at least 6 hours per day from May until November. Herd sizes ranged from 24 to 250 cows.

**Table 4:** Data structure for the cattle breeds included in multi-breed GWAS and genetic parameter estimations.

Country	Breed	No. of cows with sensor behavior data	No. of genotyped cows with sensor behavior data	No. of longitudinal sensor behavior records	No. of sensor behavior records per cow
DE	DE_DSN	69	46	22,718	329.25
PL	PL_BS	49	28	17,332	353.71
	PL_DSN	66	51	24,386	369.49
Sl	SI_Si	17	14	2,973	174.88
	SI_BS1	20	20	3,617	180.85
	SI_BS2	8	8	1,633	204.13
CH	CH_OBV	45	36	11,944	265.42
	CH_Si	45	43	12,446	276.58

DE_DSN = black and white dual-purpose (Germany); PL_BS = Brown Swiss (Poland); PL_DSN = black and white dual-purpose (Poland); Sl_Si = Simmental (Slovenia); Sl_BS = Brown Swiss (Slovenia); CH_OBV = dual-purpose Original Braunvieh (Switzerland); CH_Si = Simmental (Switzerland).

### Phenotypic data

#### Sensor traits

For the electronic recording of behavior traits, dual-purpose cows were equipped with sensors implemented in ear tags (Dutch CowManager system Agis Automatisering BV). The validation and testing phase of ear tag sensors under grazing conditions covered a period from 1 May 2016 until 31 June 2016 [68]. After one month of adaptation, ongoing analyses considered sensor data from July 2016 until March 2018 from 319 cows. Only cows with at least 30 consecutive days of sensor recording were included in the overall database. Once implemented in the cow’s left ear, the sensor system uses a 3-dimensional accelerometer to identify behavior categories based on location coordinates. The behavior categories were RMU, FEED, NACT, BACT, and HACT. In addition, the sensor systems use a digital surface temperature monitor to measure the mean hourly ET. The system detects RUM based on the typical repetitive ear movement due to chewing and regurgitation. Feeding is related to food intake, expressed through masticatory movement. The activity parameters are subcategorized into BACT, HACT and NACT. The state of BACT describes any kind of moderate ear movement resulting from walking, head shaking or other movements, which cannot be associated with the specific repetitive ear movement during RUM or FEED. High activity is due to increased BACT, e.g., during estrus periods and including mounting behavior. No activity refers to minimal ear movements, while sleeping or resting. The hourly percentage of time spent for every behavior category is transmitted through a wireless connection to a router. Afterwards, the hourly percentages for behavior traits were transformed into daily time percentages. Whenever the sensor records a certain behavior, such as RUM, it does not assign this time to another behavior trait. Additionally, to evaluate the five sensor behavior categories, two welfare indices (WEL-IP and WEL-IC) were created following the welfare quality assessment protocol^®^ [76] (Table 5). In this regard, sensor traits were assigned a score of 0, 1 or 2 according to physiological thresholds. WEL-IP was the sum of the scores from the different sensor traits. WEL-IC based on WEL-IP, but considering additional constraints as described in Table 6.

**Table 5:** Point assignment for welfare indices using the welfare quality assessment protocol® [76].

	Rumination			Feeding			Basic Active			High Active			Not Active		
	Min	Opt	Max	Min	Opt	Max	Min	Opt	Max	Min	Opt	Max	Min	Opt	Max
**Range, %/d**	< 29.2	29.2–41.7	> 41.7	< 12.5	12.5–20.8	> 20.8	< 8.3	8.3–12.5	> 12.5	< 8.3	8.3–12.5	> 12.5	< 16.7	16.7–41.7	> 41.7
**Range, h/d**	< 7	7–10	> 10	< 3	3–5	> 5	< 2	2–3	> 3	< 2	2–3	> 3	< 4	4–10	> 10
**Points**	0	2	1	0	2	1	0	2	1	1	2	0	1	2	0
**Meaning**	Al	Norm	OK	Al	Norm	OK	Al	Norm	i.h.	-	Norm	i.h.	Al	Norm	Al

Opt = optimum (normal) behavior range; Al = alarming (check animal or management); Norm = normal; OK = harmless, but not as good as Norm; i.h. = possibly in heat; the welfare index point of every observation can be calculated by summing the points for rumination, feeding, basic active, high active, and not active.

**Table 6:** Composed welfare index classes based on the welfare quality assessment protocol® [76].

Welfare index classes	Meaning	Points^a^	Criteria
**1**	Excellent	> 6 (7–10)	1) at least 1 point in every sensor trait category; 2) rumination and feeding should have 2 points according to **Table 5.**
**2**	Acceptable	5–9	
**3**	Poor (health/welfare impairment)	< 5	

^a^ Sum of welfare points across rumination, feeding, basic active, high active, and not active for each observation according to **Table 5**.

#### Production traits

Test-day records were from lactations 1 to 12 and considered the calving years from August 2015 until February 2018. Test-day MY, Fat%, Pro% and the log-transformed somatic cell count (SCS) were available for 329 cows from Germany, Poland and Switzerland. Descriptive statistics of the sensor traits, welfare indices and test-day traits are listed in Table 7.

**Table 7:** Descriptive statistics for sensor behavior and production traits.

Trait	No. of observations	No. of cows	Mean	SD	Min.	Max.
**RUM**	97,049	319	34.13	7.07	5.94	81.36
**FEED**	97,049	319	23.87	8.47	0.19	66.32
**BACT**	97,049	319	8.45	5.28	0.16	50.75
**HACT**	97,049	319	7.76	3.22	0.18	33.78
**NACT**	97,049	319	25.79	7.51	4.58	72.83
**ET**	97,049	319	24.66	4.59	2.23	38.28
**WEL-IP**	97,049	319	6.27	1.49	0	10
**WEL-IC**	97,049	319	2.04	0.42	1	3
**MY**	6,571	329	19.33	6.3	1.6	47.2
**Fat%**	6,546	329	4.1	0.67	1.84	7.98
**Pro%**	6,546	329	3.43	0.41	2.12	5.5
**SCS**	6,546	329	2.43	1.54	-1.32	10.5

RUM = rumination; FEED = feeding; BACT = basic active; HACT = high active; NACT = not active; ET = ear temperature; WEL-IP = welfare index point; WEL-IC = welfare index class; MY = milk yield (in kg); Fat% = fat percentage; Pro% = protein percentage; SCS = somatic cell score.

### Genotypes

The five dual-purpose breeds, two additional breeds from Germany (DE_DN and DE_HF) and one from Slovenia (Sl_HF) were genotyped with the *Illumina Bovine 50K Bead chip* version *2*, with the *Illumina Bovine 50K Bead chip* version *3*, and with a customized bovine 50K SNP chip (*IDB V3*), according to the Illumina Infinium assay protocol (Illumina Inc., San Diego, CA, USA). Quality controls of the genotype data were conducted using PLINK software [77], defining a minor allele frequency of 0.01 and a deviation from Hardy–Weinberg equilibrium of *p <* 0.00001. All SNPs had a call rate larger than 85%, and SNPs located on sex chromosomes were excluded. Cows with a call rate lower than 80% for all loci were excluded. Whenever the genomic relation between two animals was larger than 0.95, one animal was excluded. Sporadic missing SNPs were imputed by the BEAGLE version 3.3.2 [78]. After SNP data editing and imputation, 35,826 SNPs from 615 cows were available (Table 8), and 246 genotyped cows had sensor records.

**Table 8:** Genotype data of five cattle breeds included in PCA, WIDDE and multi-breed GWAS.

Country	Breed	No. of cows	No. of cows after SNP quality control
DE	DE_DSN	266	266
	DE_DN	20	20
	DE_HF	50	50
PL	PL_BS	34	34
	PL_DSN	59	59
Sl	Sl_Si	46	44
	Sl_BS	36	36
	Sl_HF	14	14
CH	CH_OBV	48	46
	CH_Si	48	46

DE_DSN = black and white dual-purpose (Germany); DE_DN = red and white dual-purpose (Germany); DE_HF = Holstein Friesian (Germany); PL_BS = Brown Swiss (Poland); PL_DSN = black and white dual-purpose (Poland); Sl_Si = Simmental (Slovenia); Sl_BS = Brown Swiss (Slovenia); Sl_HF = Holstein Friesian (Slovenia); CH_OBV = dual-purpose Original Braunvieh (Switzerland); CH_Si = Simmental (Switzerland).

The data used in the present study is available as supplementary file S1 File.

### Population structure and breed assignment

PCA was conducted to account for potential population stratification prior to GWAS and to explore the genetic diversity of the European cow dataset. PCA based on the genomic relationship matrix was generated in GCTA [79]. In a second step, a breed assignment analysis was conducted using the WIDDE program [16]. The WIDDE cattle database contained over 750,000 SNPs from 3,951 individuals, which belong to 129 different populations [16]. The broad variety of local cattle populations in WIDDE represents the bovine genetic diversity and covers the three main cattle groups, i.e., European and African taurine (*Bos taurus*) as well as zebus (*Bos indicus*) [16]. The allele proximity between the genotyped populations from this study and the populations represented in the world reference dataset in WIDDE were estimated using supervised clustering [16]. A convergence criterion of 0.01 for log-likelihood values was defined when calculating the percentage of ancestry proportions between each genotyped cow and the 129 populations from the WIDDE world reference dataset.

### Genetic parameter estimations

For the estimation of genetic parameters, genomic and pedigree relationship matrices were combined. In additional analyses, only the pedigree relationship matrix was considered. The pedigree consisted of 8,798 animals and was traced back as far as possible. The oldest ancestors in the pedigree were born in 1944 for Germany, in 1981 for Poland, in 1990 for Slovenia, and in 1917 for Switzerland. Variance components of sensor traits were estimated via univariate animal models using the AIREML procedure, as implemented in the DMU software package [80]. The statistical model (1) in matrix notation was defined as follows:

$$y=Xb+Z_{1}a+Z_{2}p+e$$ (1)

where **y** was the observation vector for sensor traits and indices (RUM, FEED, NACT, BACT, HACT, ET, WEL-IP and WEL-IC); **b** was the vector of fixed effects including the combined breed-farm effect, the year-month effect for the measuring date, and the age of cows as a fixed linear regression; **a** was the vector for additive genetic effects; **p** was the vector for permanent environmental effects for the cows with repeated measurements; **e** was the vector of random residual effects, and **X**, **Z**_**1**_, and **Z**_**2**_ were incidence matrices for **b**, **a**, and **p**, respectively. The assumed variance-covariance structure was **a** ~ *N* (0, ${\text{K}\sigma }_{\text{a}}^{\text{2}}$), where $\sigma_{\text{u}}^{\text{2}}$ was the genetic variance, **K** was the **A** matrix, or the combined **H** matrix when blending **A** and the weighted genomic relationship matrix (**G**_**w**_) [81]. **G**_**w**_ was calculated as follows:

$$G_{w}=\left(0.95\times G+0.05\times A_{22}\right)$$

where **A**_**22**_ is the submatrix of the pedigree-based relationship matrix for genotyped animals. Estimated breeding values (EBV) from model 1 and consideration of the **A** matrix were the databases for the calculation of DRP according to Garrick et al. [82]. Only animals with a DRP weight larger than 0.2 were considered for the ongoing GWAS [83].

The genetic-statistical model (2) used for test-day production traits and SCS based on the **A** matrix was defined as follows:

$$y=Xb+Z_{1}a+Z_{2}p+e$$ (2)

where **y** was the observation vector for MY, Fat%, Pro%, and SCS; **b** was the vector of fixed effects including the breed-farm and calving-year-season effects, and the lactation curve modeled via Legendre polynomials of order three for days in milk; **a** was the vector for additive genetic effects based on the **A** matrix; **p** was the vector for permanent environmental effects for the cows with repeated measurements; **e** was the vector of random residual effects, and **X**, **Z**_**1**_, and **Z**_**2**_ were the incidence matrices for **b**, **a**, and **p**, respectively. Again, EBV were de-regressed to obtain DRP for test-day production traits and SCS.

### Multi-breed GWAS

Single-trait multi-breed GWAS was performed using the software package GCTA [79]. In this regard, we considered the leave-one-chromosome-out (*loco*) option. Dependent variables (i.e., our phenotypes) were MEAN and DRP. For testing single-locus SNP effects, the following statistical model (3) was used:

y=Xb+Wg+Zu+e (3)

where **y** was the vector of DRP or MEAN for RUM, FEED, NACT, BACT, HACT, ET, WEL-IP, and WEL-IC, as well as DRP for production traits; b was a vector of fixed effects considering only the overall mean for DRP and additionally the breed-farm effect for MEAN; *g* was the vector for additive fixed effects of the candidate SNP; **u** was the vector for polygenic effects considering all SNPs but excluding SNPs from the chromosome where the candidate SNP was located; and **e** was the vector of random residual effects; **X**, **W**, and **Z** were incidence matrices for **b**, **g**, and **u**, respectively. The Bonferroni threshold for SNP associations was *p*_Bonf_ = 0.05/(number of SNP) = 0.05/35,826 = 4.47 x 10^−7^. The FDR as introduced by Benjamini and Hochberg [84] was a further significance threshold for genome-wide associations. The FDR to detect candidate SNPs for behavior traits and test-day production traits was set to 20%.

### Candidate gene annotation

The associated potential candidate genes were identified via a gene-based test in GCTA and applying the *fastBAT* option [48]. The database (version UMD3.1), including gene locations and start and end positions for the bovine genes, was downloaded from Ensembl [50]. A total of 24,616 gene ID entries were originally available in the database, but only 17,545 genes on chromosomes 1 to 29 were included in further analyses (i.e., exclusion of pseudogenes according to [76,85,86]). In the first step, all SNPs from the GWAS were mapped to the genes, considering a window of 50 kb upstream and 50 kb downstream from the genes. Subsequently, *P-values* considering the set of SNPs within the window were used simultaneously for candidate gene detection. The *P-values* of genes were adjusted according to FDR (significance threshold < 20%). In the last step, physiological functions and positions of candidate genes were inferred based on information from the Ensembl [50], NCBI [87], UniProt [88] and Genecard [61] databases.

### Causal associations

In epidemiology, Mendelian randomization uses genetic variants as instrumental variables to test for the causal inference between an exposure and an outcome [89]. Hence, we assume an instrumental variable z, representing the SNP genotype. The exposure x considered one of the behavior traits, and the outcome y was the test-day productivity or SCS. Assuming uncorrelated *z* and uncorrelated residuals when regressing *y* on *x* and cov(z,x)≠0, the regression coefficient of ${\hat{b}}_{yx}$ was [90]:

$${\hat{b}}_{yx}=\frac{cov\left(z,\;y\right)}{cov\left(z,\;x\right)}=\frac{\frac{cov\left(z,y\right)}{var\left(z\right)}}{\frac{cov\left(z,x\right)}{var\left(z\right)}}=\frac{{\hat{b}}_{yz}}{{\hat{b}}_{xz}}$$ (4)

where ${\hat{b}}_{yz}$ and ${\hat{b}}_{xz}$ were the estimated SNP effects from GWAS when using *y* and *x* as phenotypes, respectively. The variance of ${\hat{b}}_{yx}$ was:

$$var\left({\hat{b}}_{yx}\right)=\frac{var\left(e_{yx}\right)}{n\cdot var\left(x\right)\cdot R_{xz}^{2}}=\frac{var\left(y\right)\cdot \left(1-\rho_{xy}^{2}\right)}{n\cdot var\left(x\right)\cdot \frac{2\cdot p\cdot \left(1-p\right)\cdot {\hat{b}}_{xz}^{2}}{var\left(x\right)}}=\frac{var\left(y\right)\cdot \left(1-\rho_{xy}^{2}\right)}{n\cdot 2\cdot p\cdot \left(1-p\right)\cdot {\hat{b}}_{xz}^{2}}$$ (5)

where $var(e_{xy})$ was the residual variance when fitting *x* as a fixed regression to explain *y*; n was the sample size; var(x) was variance of trait *x*; and $R_{xz}^{2}$ was the proportion of variance *x* explained by *z*, which equaled $\frac{2∙p∙\left(1-p\right)∙{\hat{b}}_{xz}^{2}}{var(x)}$. In Eq (5), var(y) was the variance of trait *y*; $\rho_{xy}^{2}$ was the squared correlation between trait *x* and trait *y*; *p* was the allele frequency, and ${\hat{b}}_{xz}^{2}$ was the squared SNP effect estimate from GWAS for trait *x*. The test statistic $T_{MR}\;=\;\frac{{\hat{b}}_{yx}^{2}}{var({\hat{b}}_{yx})}$ followed $\chi_{1}^{2}$ [91], which was used to test the significance of ${\hat{b}}_{yx}$.

To fulfill the precondition of cov(z,x)≠0, 445 homologous genes in the human and bovine genome that were involved in the biological process of behavior were searched and downloaded from AmiGO2, a Gene Ontology database [85,86]. A total of 1,011 SNPs within a window of 50 kb up- and downstream of the 445 homologous genes were considered.

Afterwards, the GWAS estimates for the 1,011 SNP were transmitted into a self-modified version of the GSMR package [92] in R to calculate the variance of ${\hat{b}}_{yx}$ for a small sample size. The aforementioned SNP was filtered according to the following criteria: 1) *P-value* of the SNP lower than 0.05 to meet the assumption of cov(z,x)≠0; and 2) LD between SNP lower than 0.25 to prune highly correlated SNPs. After filtering, the number of SNPs for behavior traits and welfare indices varied between 36 (for BACT) and 64 (for RUM).

## Supporting information

S1 Fig Manhattan plot and Q-Q plot from GWAS based on the mean (MEAN) and de-regressed proofs (DRP) of daily not active time. The red line is the significance threshold line for the Bonferroni correction of 5%, and the green dots represent significant SNP according to the false discovery rate of 20%.
(PDF)

S2 Fig Manhattan plot and Q-Q plot from GWAS based on de-regressed proof of daily rumination time. The red line is the significance threshold line for the Bonferroni correction of 5%, and the green dots represent significant SNP according to the false discovery rate of 20%.
(PDF)

S3 Fig Manhattan plot and Q-Q plot from GWAS based on de-regressed proof of daily feeding time. The red line is the significance threshold line for the Bonferroni correction of 5%, and the green dots represent significant SNP according to the false discovery rate of 20%.
(PDF)

S4 Fig Manhattan plot and Q-Q plot from GWAS based on de-regressed proof of test-day fat percentage. The red line is the significance threshold line for the Bonferroni correction of 5%, and the green dots represent significant SNP according to the false discovery rate of 20%.
(PDF)

S1 Table Potential candidate genes associated with animal behavior traits. RUM = rumination; FEED = feeding; BACT = basic active; BTA = Bos taurus chromosome; DRP = de-regressed proof; MEAN = mean of observations; functions derived from Ensembl^1^, NCBI^2^, UNIPROT^3^, and GeneCard^4^.
(DOCX)

S2 Table Genetic (above diagonal) and phenotypic (below diagonal) correlations among sensor behavior. Correlations estimated from bivariate models with the same fixed and random effects as model (1) and standard errors in brackets. RUM = rumination; FEED = feeding; BACT = basic active; HACT = high active; NACT = not active; ET = ear temperature; WEL-IP = welfare index point; WEL-IC = welfare index class; nc = did not converge.
(DOCX)

S1 File Raw phenotypes, genotypes, pedigrees, and values to build Figs 1 and 2 are available in the compressed file. (ZIP)
