# Supplementary figures and images for: Multi-breed genome-wide association studies across countries for electronically recorded behavior traits in local dual-purpose cows
Source: PLoS One. 2019 Oct 30;14(10):e0221973. doi: 10.1371/journal.pone.0221973 (PMC6821105; doi:10.1371/journal.pone.0221973)

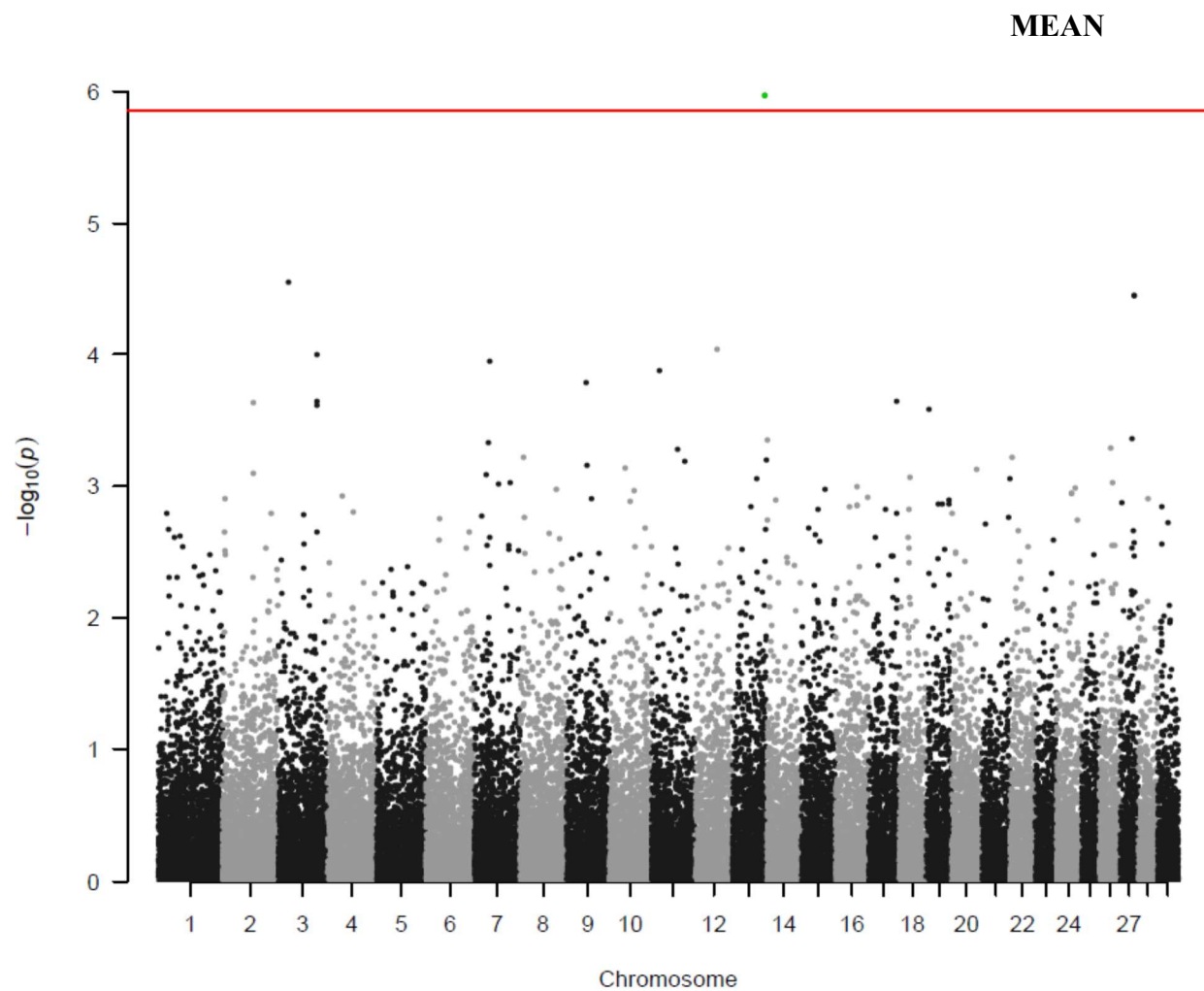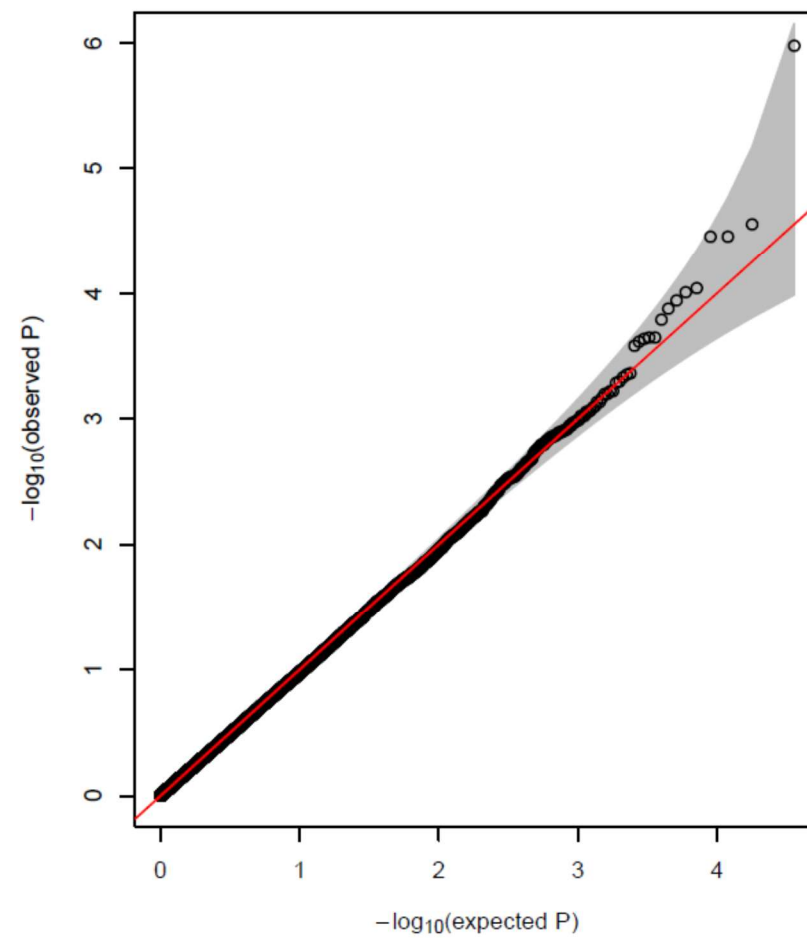

## DRP

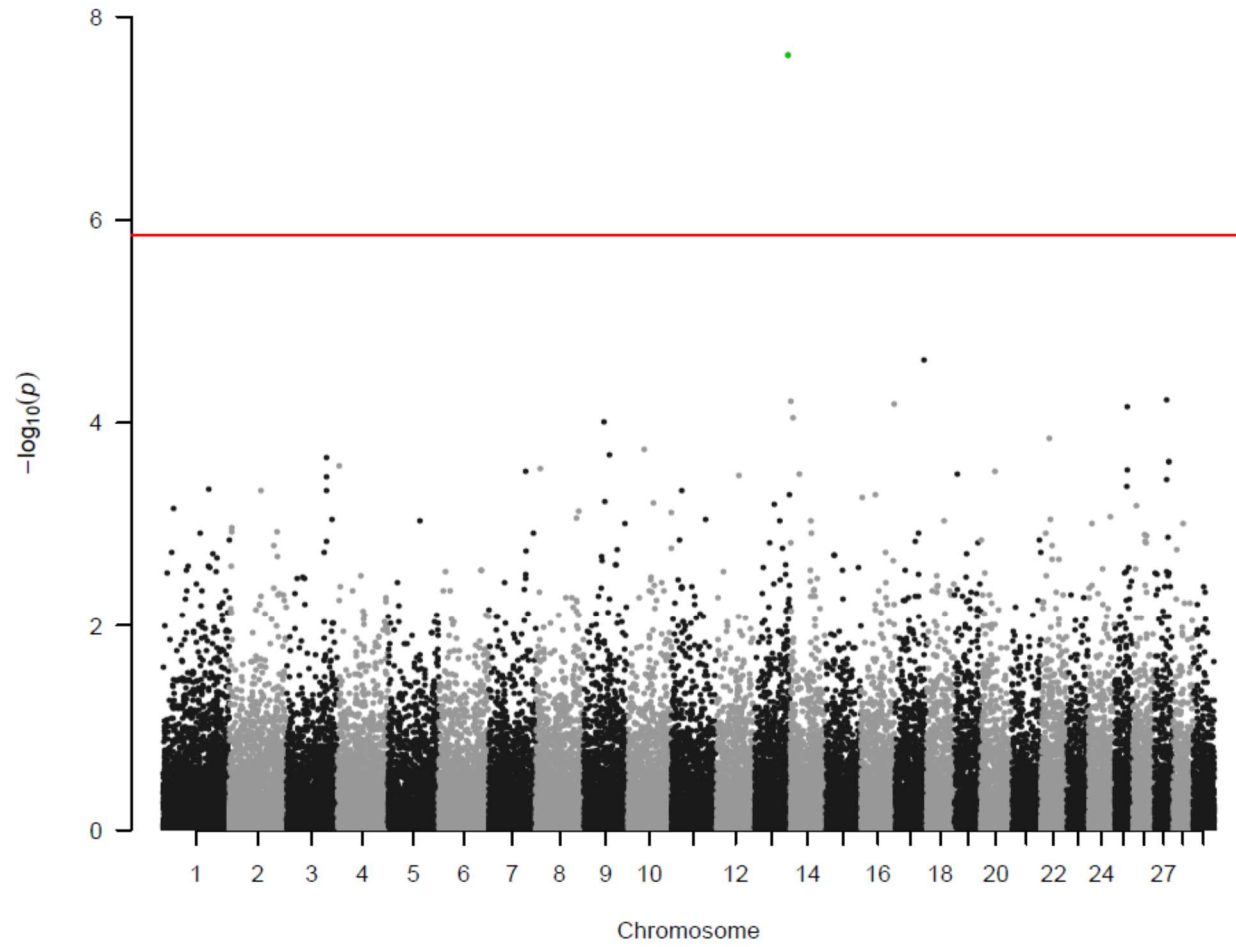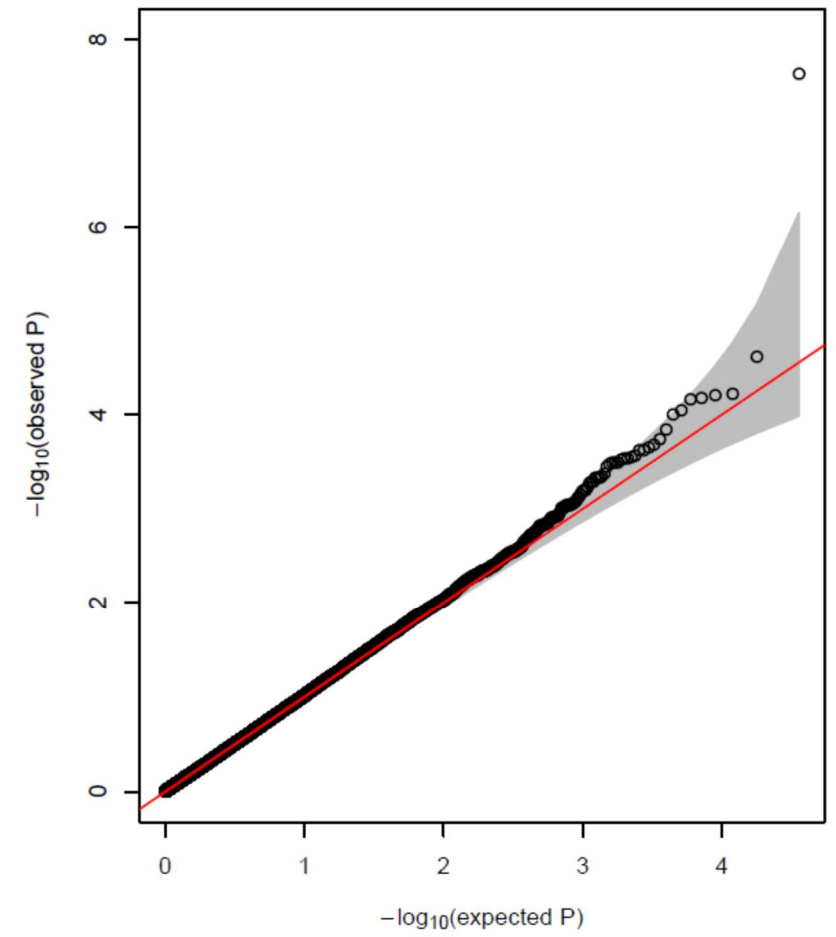

Supplement: S1 Fig — The red line is the significance threshold line for the Bonferroni correction of 5%, and the green dots represent significant SNP according to the false discovery rate of 20%. (PDF) [file pone.0221973.s001.pdf]

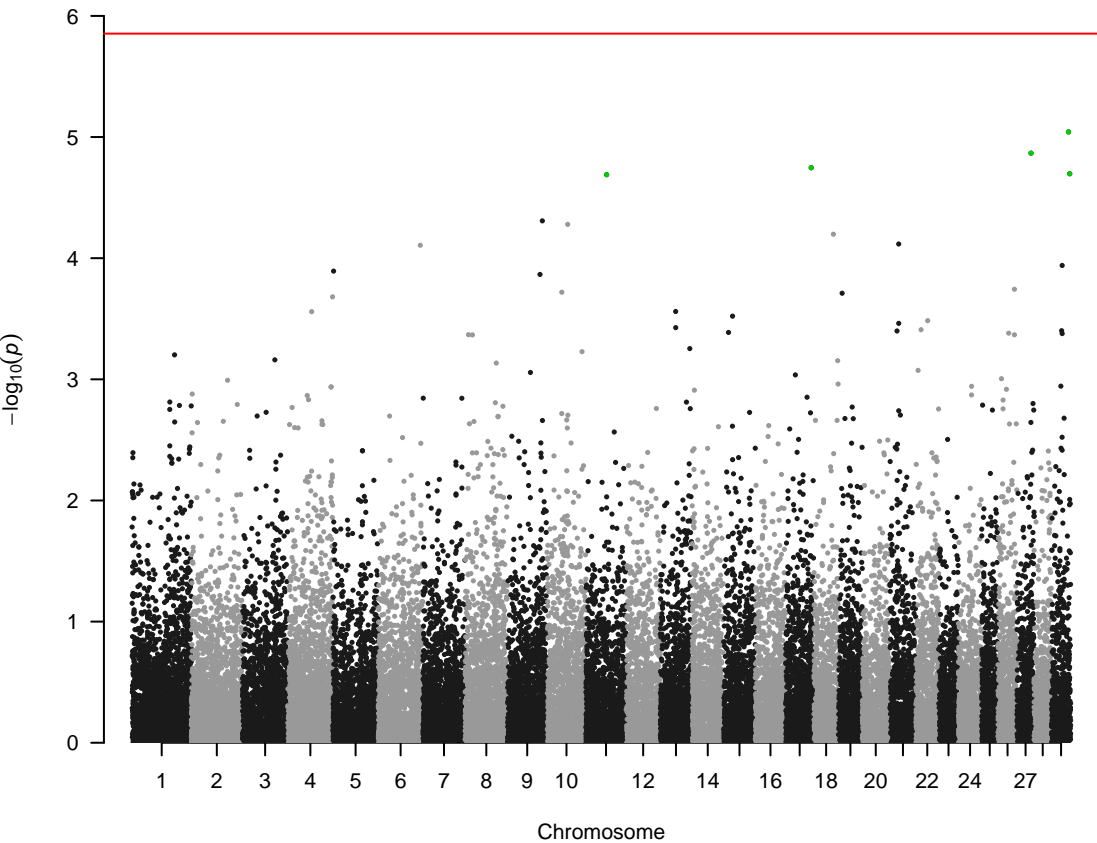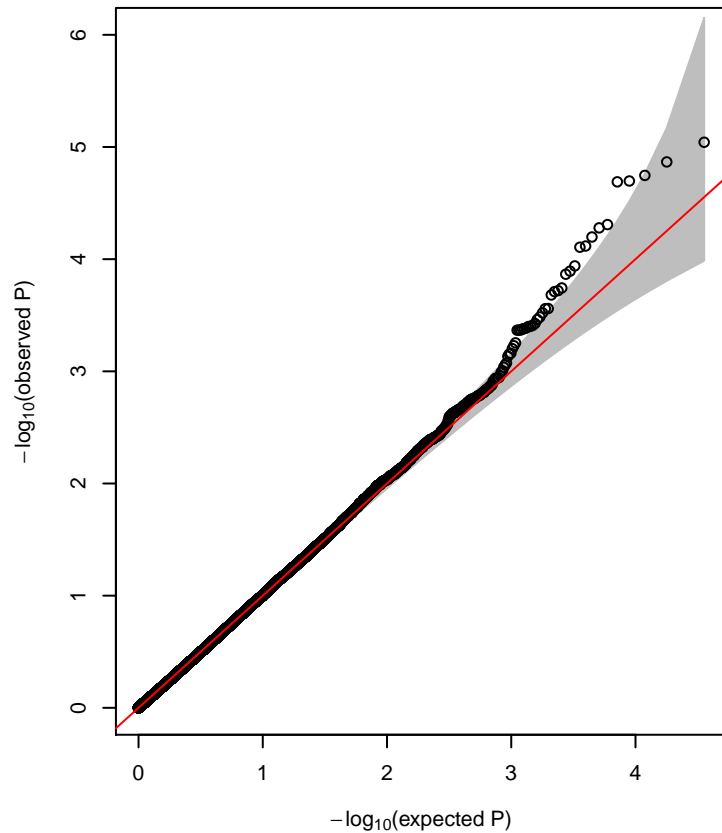

Supplement: S2 Fig — The red line is the significance threshold line for the Bonferroni correction of 5%, and the green dots represent significant SNP according to the false discovery rate of 20%. (PDF) [file pone.0221973.s002.pdf]

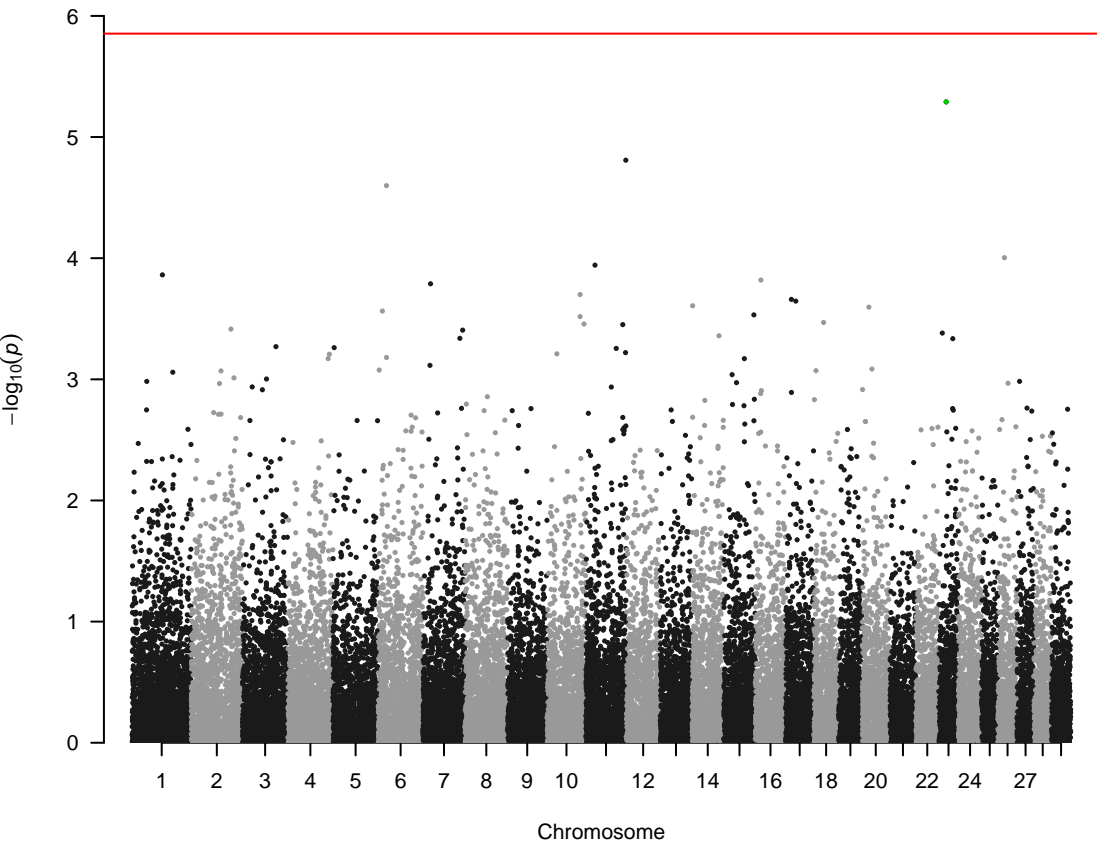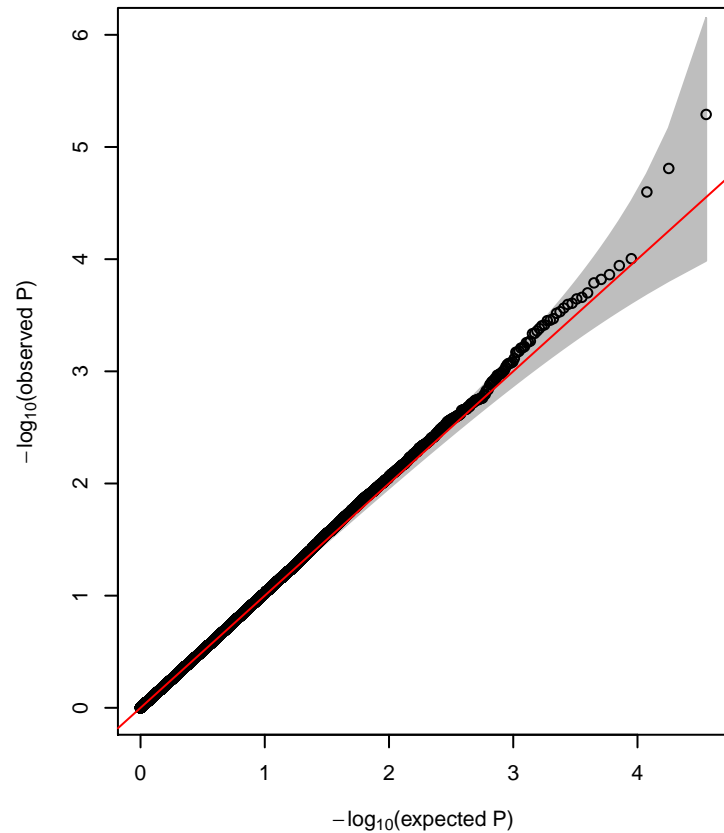

Supplement: S3 Fig — The red line is the significance threshold line for the Bonferroni correction of 5%, and the green dots represent significant SNP according to the false discovery rate of 20%. (PDF) [file pone.0221973.s003.pdf]

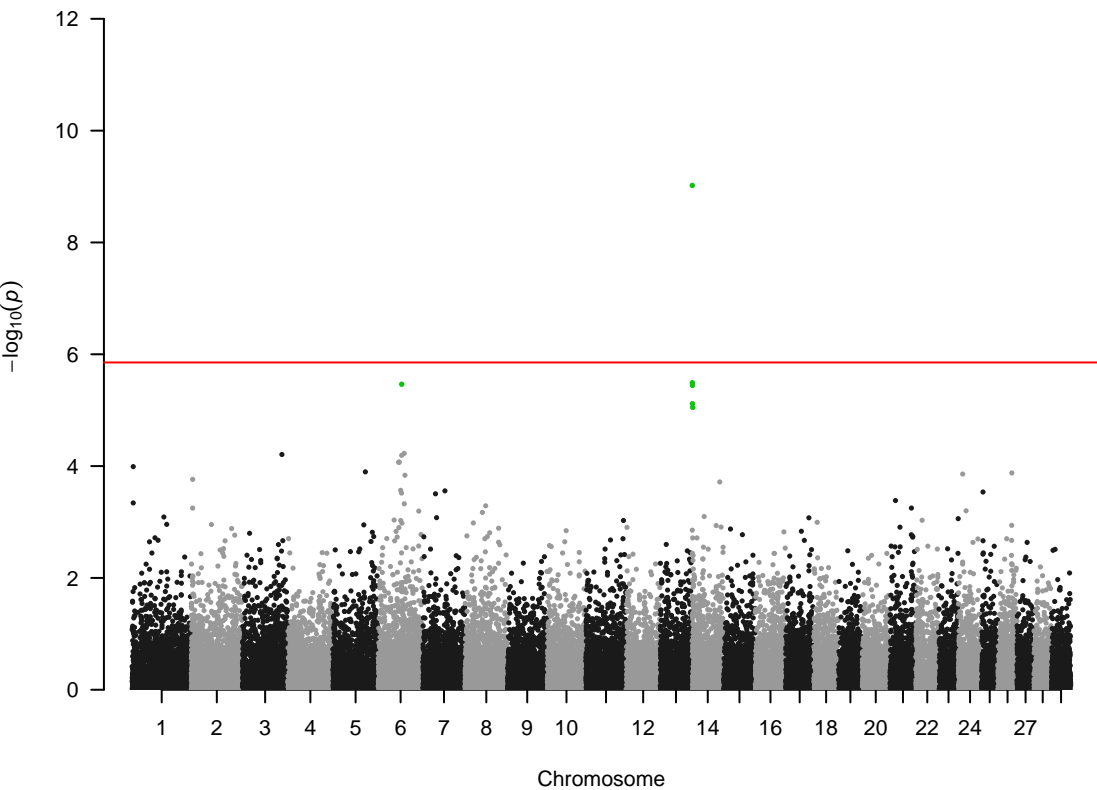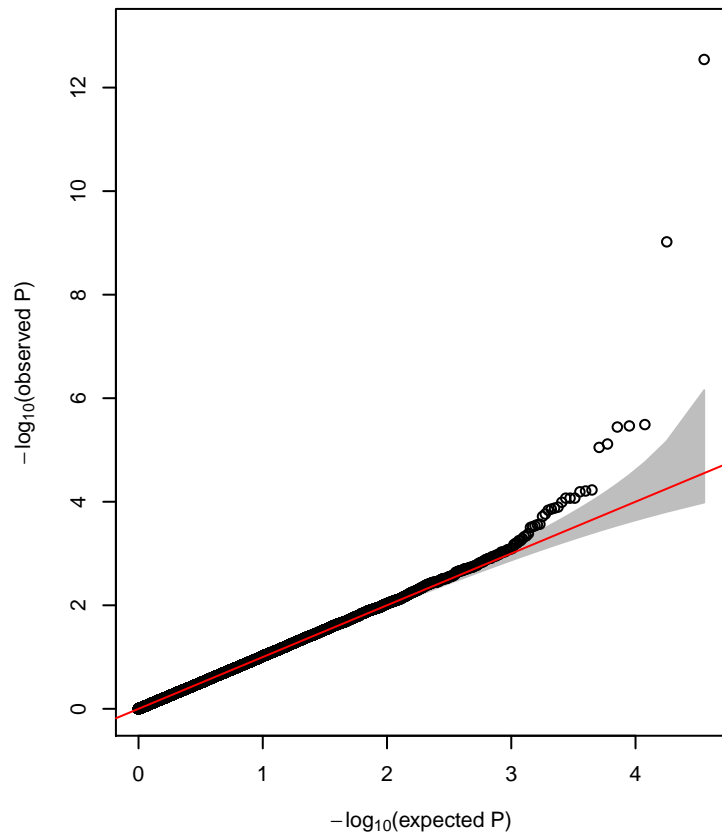

Supplement: S4 Fig — The red line is the significance threshold line for the Bonferroni correction of 5%, and the green dots represent significant SNP according to the false discovery rate of 20%. (PDF) [file pone.0221973.s004.pdf]
