# Supplementary material for: Multi-breed genome-wide association studies across countries for electronically recorded behavior traits in local dual-purpose cows
Source: PLoS One. 2019 Oct 30;14(10):e0221973. doi: 10.1371/journal.pone.0221973 (PMC6821105; doi:10.1371/journal.pone.0221973)
Supplement: S1 Table — RUM = rumination; FEED = feeding; BACT = basic active; BTA = Bos taurus chromosome; DRP = de-regressed proof; MEAN = mean of observations; functions derived from Ensembl1, NCBI2, UNIPROT3, and GeneCard4. (DOCX) [file pone.0221973.s005.docx]

**S1 Table.** **Potential candidate genes associated with animal behaviour traits.**

| Trait | Phenotype | BTA | SNP | Candidate gene | Biological process | Start of Gene |
| --- | --- | --- | --- | --- | --- | --- |
| RUM | DRP | 21 | BTB-00811262 | *BTBD1* | Neurogenesis^1^; regulation of proteolysis^1^; proteasome-mediated ubiquitin-dependent protein catabolic process^1^; key protein of balance between adipogenesis and myogenesis^2^; expressed particularly in skeletal muscle^2^ | 25467798 |
|  | DRP | 27 | ARS-BFGL-NGS-67948 | *THAP1* | Negative regulation of transcription by RNA polymerase II^1^; endothelial cell proliferation^1^; transcription; DNA-templated^1^; regulation of mitotic cell cycle^1^ | 37234389 |
|  | DRP | 27 | ARS-BFGL-NGS-67948 | *RNF170* | Mutations in this gene are associated with autosomal dominant sensory ataxia in human^2^ | 37246976 |
|  | DRP; MEAN | 29 | ARS-BFGL-NGS-118751 | *RPS6KB2* | Protein phosphorylation^1^; signal transduction^1^; Protein kinase B signalling^1^; positive regulation to translational initiation^1^ | 45965769 |
|  | DRP; MEAN | 29 | ARS-BFGL-NGS-118751 | *PTPRCAP* | Defence response^3^ | 45972077 |
|  | DRP; MEAN | 29 | ARS-BFGL-NGS-118751 | *CORO1B* | Actin cytoskeleton organization^1^; actin filament branching^3^; cellular response to platelet-derived growth factor stimulus^3^; endothelial cell chemotaxis^3^; negative regulation of smooth muscle cell chemotaxis^4^; wound healing^3^ | 45974636 |
|  | DRP; MEAN | 29 | ARS-BFGL-NGS-118751 | *GPR152* | G-protein coupled receptor activity^1^ | 45985247 |
|  | DRP; MEAN | 29 | ARS-BFGL-NGS-118751 | *CaBP4* | Visual perception^1^; phototransduction^1^; photoreceptor cell morphogenesis^1^; retinal cone cell development^1^; retinal bipolar neuron differentiation ^1^ | 45988625 |
|  | DRP; MEAN | 29 | ARS-BFGL-NGS-118751 | *TMEM134* | Atherosclerosis and obesity in adults [61] | 45997538 |
|  | DRP; MEAN | 29 | ARS-BFGL-NGS-118751 | *AIP* | Protein folding^1^; protein targeting to mitochondrion^1^; xenobiotic metabolic process ^1^; protein maturation by protein folding^1^ | 46012079 |
|  | DRP; MEAN | 29 | ARS-BFGL-NGS-118751 | *PITPNM1* | Diseases associated include retinal degeneration and hypopyon^4^ | 46018812 |
|  | DRP | 29 | ARS-BFGL-NGS-118384 | *ENSBTAG0000*  *0000776* | G-protein coupled receptor signalling pathway^1^ | 48988149 |
|  | DRP | 29 | ARS-BFGL-NGS-61470 | *MRGPRG* | G-protein coupled receptor signalling pathway^1^ | 49026020 |
| BACT | MEAN | 19 | Hapmap48998-BTA-104140 | *PPM1E* | Negative regulation of protein kinase activity^1^; protein dephosphorylation^1^; cellular response to drug^1^; peptidyl-threonine dephosphorylation^1^; positive regulation of stress fibre assembly^1^ | 10143539 |
| FEED | DRP | 11 | ARS-BFGL-NGS-27581 | *STXBP1* | Platelet degranulation^1^; vesicle docking involved in exocytosis^1^; neurotransmitter secretion^1^; neuromuscular synaptic transmission^3^; axon target recognition^3^; negative regulation of neuron apoptotic process^3^ | 98325785 |
|  | DRP | 11 | ARS-BFGL-NGS-27581 | *CFAP157* | Sperm axoneme assembly^3^ | 98400784 |
|  | DRP | 11 | ARS-BFGL-NGS-27581 | *PTRH1* | - | 98406893 |
|  | DRP | 11 | ARS-BFGL-BAC-15613 | *TOR2A* | Chaperone mediated protein folding requiring cofactor^1^; protein homooligomerization^1^ | 98425082 |
|  | DRP | 11 | ARS-BFGL-NGS-13735 | *LCN8* | Response to hormone^1^ | 106391056 |
|  | DRP | 11 | ARS-BFGL-NGS-13735 | *LCN15* | Lipid metabolic process^1^; transport^1^ | 106397537 |
|  | DRP | 11 | ARS-BFGL-NGS-21943 | *PPP1R26* | Negative regulation of phosphatase activity^1^ | 106462138 |
|  | DRP | 23 | ARS-BFGL-NGS-80066 | *SLC25A27* | Mitochondrial transport^3^; regulation of mitochondrial membrane potential^3^; response to cold^3^; long chain fatty acids uptake gene [65] | 19860795 |

RUM = rumination; BACT = basic active; FEED = feeding; BTA = Bos taurus chromosome; DRP = de-regressed proof; MEAN = mean of observations; Functions derived from Ensembl^1^, NCBI^2^, UNIPROT^3^, and GeneCard^4^.
