# Supplementary material for: Multi-breed genome-wide association studies across countries for electronically recorded behavior traits in local dual-purpose cows
Source: PLoS One. 2019 Oct 30;14(10):e0221973. doi: 10.1371/journal.pone.0221973 (PMC6821105; doi:10.1371/journal.pone.0221973)
Supplement: S2 Table — Correlations estimated from bivariate models with the same fixed and random effects as model (1) and standard errors in brackets. RUM = rumination; FEED = feeding; BACT = basic active; HACT = high active; NACT = not active; ET = ear temperature; WEL-IP = welfare index point; WEL-IC = welfare index class; nc = did not converge. (DOCX) [file pone.0221973.s006.docx]

**S2 Table. Genetic (above diagonal) and phenotypic (below diagonal) correlations among sensor behavior.**

|  | RUM | FEED | BACT | HACT | NACT | ET | WEL-IP | WEL-IC |
| --- | --- | --- | --- | --- | --- | --- | --- | --- |
| RUM |  | 0.31 (0.69) | -0.78 (0.64) | -0.18 (0.51) | -0.34 (0.58) | nc | nc | nc |
| FEED | -0.13 (0.02) |  | -0.67 (0.29) | -0.02 (0.19) | -0.85 (0.09) | -0.33 (0.28) | -0.37 (0.28) | nc |
| BACT | -0.50 (0.01) | -0.40 (0.01) |  | 0.04 (0.30) | 0.49 (0.37) | -0.05 (0.48) | 0.61 (0.49) | nc |
| HACT | -0.28 (0.02) | -0.08 (0.02) | 0.18 (0.02) |  | -0.33 (0.20) | 0.30 (0.30) | 0.28 (0.28) | -0.06 (0.25) |
| NACT | -0.33 (0.02) | -0.59 (0.01) | 0.06 (0.02) | -0.22 (0.02) |  | 0.29 (0.35) | 0.39 (0.35) | nc |
| ET | nc | -0.16 (0.02) | 0.07 (0.02) | 0.11 (0.02) | -0.01 (0.02) |  | 0.23 (0.43) | -0.11 (0.39) |
| WEL-IP | nc | -0.14 (0.01) | 0.06 (0.01) | 0.05 (0.01) | -0.09 (0.01) | 0.07 (0.01) |  | -0.87 (0.08) |
| WEL-IC | nc | nc | nc | 0.02 (0.01) | nc | -0.05 (0.01) | -0.77 (0.00) |  |

RUM = rumination; FEED = feeding; BACT = basic active; HACT = high active; NACT = not active; ET = ear temperature; WEL-IP = welfare index point; WEL-IC = welfare index class; nc = did not converge.
